# Case Report: Coadministration of TNF-α blockade as an effective adjunctive strategy in the treatment of ANCA-negative EGPA with refractory peripheral neuropathy

**DOI:** 10.3389/fphar.2025.1555377

**Published:** 2025-07-09

**Authors:** Dabin Tang, Yubao Jiang, Shuo Yang, Jiaxin Wei, Jingjing Xie, Jianyong Zhang

**Affiliations:** ^1^ The Department of Rheumatology, The fourth Clinical Medical College of Guangzhou University of Chinese Medicine, Shenzhen, Guangdong, China; ^2^ The Department of Rheumatology, Shenzhen Traditional Chinese Medicine Hospital, Shenzhen, Guangdong, China

**Keywords:** eosinophilic granulomatosis with polyangiitis, eosinophilia, refractory peripheral neuropathy, TNF-α blockade, etanercept

## Abstract

Eosinophilic granulomatosis with polyangiitis (EGPA) is characterized by abnormal eosinophilia and extravascular eosinophilic granuloma formation. Conventional treatments combining glucocorticoids and immunosuppressants can alleviate the condition in most patients with EGPA. However, the efficacy of blocking tumor necrosis factor (TNF)-α-mediated inflammatory reactions in managing EGPA remains controversial. Here, we report a case of refractory EGPA that did not respond to glucocorticoid pulse therapy and adequate cyclophosphamide. The disease was ultimately controlled through the coadministration of etanercept.

## 1 Introduction

Eosinophilic granulomatous polyangiitis (EGPA) is a rare vasculitis distinguished by peripheral blood eosinophilia and extravascular eosinophilic granuloma formation. Corticosteroids serve as the foundational therapy for EGPA, swiftly diminishing eosinophil levels in the blood and managing the manifestations of vasculitis. However, managing steroid tapering can be challenging in cases of uncontrollable asthma or extrapulmonary manifestations. Prolonged steroid use may lead to significant side effects, necessitating the use of immunosuppressive agents in combination during the induction and remission treatment of EGPA (Emmi et al., 2023).

Peripheral neuropathy is common in most patients with EGPA and often results in impaired limb function. Timely and effective treatment may mitigate this damage. The role of tumor necrosis factor (TNF)-α in the pathogenesis of EGPA is debated. On one hand, TNF-α can decrease IL-5 levels and eosinophilia, shifting inflammation from a neutrophil to an eosinophil bias (Fei et al., 2011); however, TNF-α blockade has shown no efficacy in treating anti-neutrophil cytoplasmic antibody (ANCA)-associated vasculitis (Group. WsGETWR, 2005). Conversely, TNF-α can delay eosinophil apoptosis, and blocking TNF-α can reduce eosinophil counts (Kankaanranta et al., 2014), potentially making it a viable treatment option for EGPA.

## 2 Case presentation

A 40-year-old man presented with a history of chronic rhinitis and nasal polyps diagnosed 10 years prior, and asthma diagnosed 2 years prior, for which he received medium-dose inhaled corticosteroids. Five days before hospital admission, he experienced a fever (up to 38.5°C), frequent cough with minimal sputum production, exertional dyspnea, persistent nasal congestion, paresthesia in all four limbs, and arthralgia in both wrists. Four days before admission, he developed progressive pain in his left upper and lower extremities, occurring in the afternoon or evening and lasting approximately 2–3 h. The patient was admitted due to dyspnea and severe pain. On the day of admission, auscultation revealed wheezing in the bilateral lung fields, and neurological examination showed no abnormalities. His vital signs included a body temperature of 38.1°C, blood pressure of 125/95 mmHg, pulse rate of 115 beats per minute, respiration rate of 20 breaths per minute, and oxygen saturation of 95% on room air. Arterial blood gas analysis on room air indicated a pH of 7.408, a partial pressure of carbon dioxide of 41 mmHg, and a partial pressure of oxygen of 74.1 mmHg.

**FIGURE 1:**
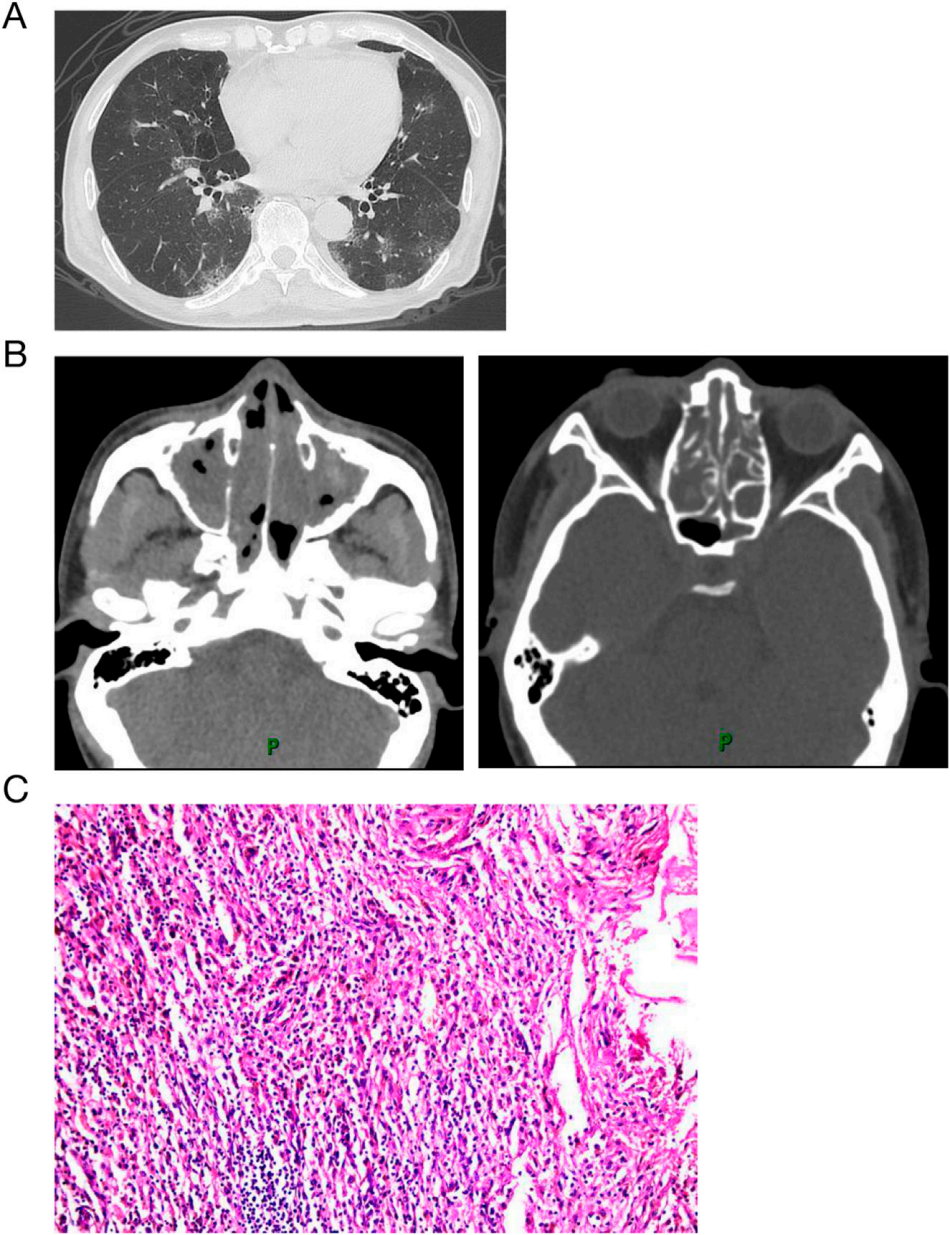
Computed tomography and histology on admission. Computed tomography revealed ground-glass opacities and consolidation in the bilateral lung fields **(A)**, along with paranasal sinusitis **(B)**. Biopsies from lung with hematoxylin and eosin staining show eosinophilic infltration.

Laboratory findings revealed a white blood cell count of 19,580/μL with 27.1% eosinophils, hemoglobin level of 111 g/dL, C-reactive protein level of 27.8 mg/dL, IgE level of 705 IU/mL, albumin concentration of 26.7 g/L, and rheumatoid factor of 24.8 IU/mL (normal range: 0–14 IU/mL). Tests for myeloperoxidase-antineutrophil cytoplasmic antibody (ANCA), proteinase-3-ANCA, erythrocyte sedimentation rate, and antinuclear antibodies were negative. Computed tomography revealed ground-glass opacities and consolidation in the bilateral lung fields, along with paranasal sinusitis (Figures 1A,B). Electromyography indicated electrophysiological signs of sensory and motor nerve fiber injury in all four limbs. The electrocardiogram suggested sinus tachycardia, rightward deviation of the cardiac axis, and counterclockwise rotation of the cardiac potentials. Bone marrow smear suggested active myeloproliferative activity and eosinophilia. Cerebrospinal fluid examination showed no abnormalities. Transbronchial lung biopsies revealed eosinophilic infiltration (Figure 1C). The patient was diagnosed with eosinophilic granulomatosis with polyangiitis (EGPA) based on the American College of Rheumatology diagnostic criteria.

**FIGURE 2:**
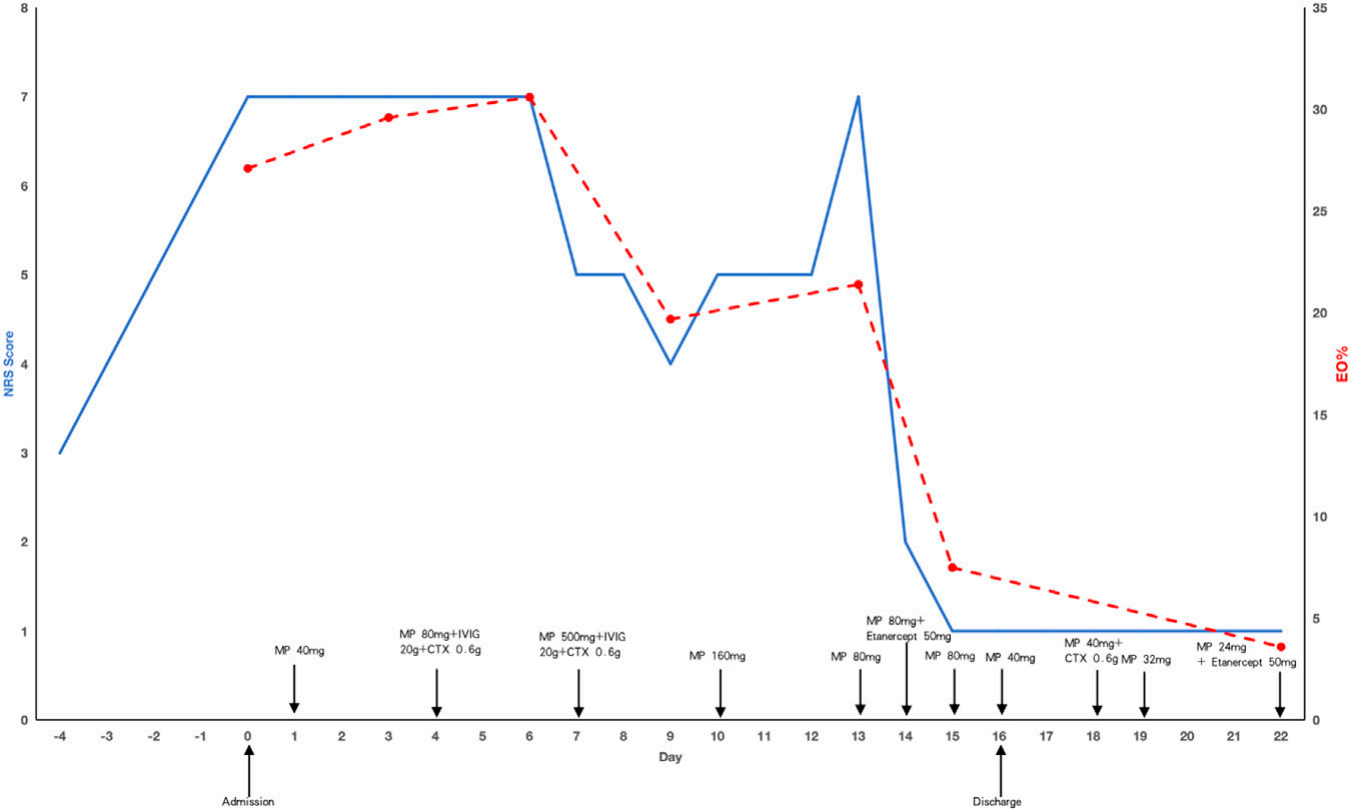
The medication record and the clinical course of the patient. The solid blue line represents changes in NRS scores, the red dotted line represents the changes in eosinophil proportion. MP: methylprednisolone, CTX: cyclophosphamide, NRS: numeric rating scale, Eo: eosinophil.

During hospitalization prior to treatment, the patient experienced severe pain in his left upper and lower extremities for two to 3 h each afternoon and evening. The numerical rating scale (NRS) was utilized to measure pain intensity, with scores of 7, occasionally peaking at 8. Initially, the patient was administered an intravenous dose of methylprednisolone at 40 mg/day. After 3 days of treatment, there was no improvement in either the pain or the eosinophilia. Consequently, the methylprednisolone dosage was increased to 80 mg/day, and immunoglobulin at 20 g/day along with cyclophosphamide at 0.6 g every 2 weeks were coadministered. Despite these adjustments, the patient remained unresponsive to the treatment. A regimen of 500 mg/day methylprednisolone pulse therapy combined with 20 g/day immunoglobulin for 3 days resulted in a reduction of eosinophils and slight improvement in peripheral neuropathy, with the NRS score decreasing to 4. However, when methylprednisolone was tapered to 80 mg/day (following 160 mg/day for 3 days), eosinophil rose significantly, peripheral neuropathy worsened, and the NRS score increased to 7. Consequently, etanercept at 50 mg weekly was administered, leading to remarkable improvement in neuropathy that same day, with the NRS score dropping to 1 and paresthesia significantly alleviated. A blood test the following day showed a dramatic decrease in eosinophils and discharge was arranged. The patient was then prescribed a regimen of tapered methylprednisolone, cyclophosphamide 0.6 g biweekly and etanercept 50 mg weekly. Despite treatment, the patient was discharged with impaired left hand function, characterized by muscle atrophy, decreased muscle strength, and an inability to make a tight fist. Three months later, etanercept was discontinued, maintenance therapy with methylprednisolone 4 mg every other day and methotrexate 10 mg weekly was initiated. After nearly 1 year of rehabilitation training and acupuncture treatment, the function of the left hand was almost restored. A two-year follow-up revealed no recurrence of symptoms. The medication record and the clinical course of the patient was shown in Figure 2.

## 3 Discussion

EGPA is a granulomatous vasculitis typically preceded by asthma, eosinophilia. Peripheral neuropathy is common in most patients, which is often characterized by multiple mononeuritis and an asymmetric distribution. Although peripheral neuropathy does not reduce life expectancy, it can result in limb insufficiency sequelae that significantly diminish quality of life (Wolf et al., 2010). Therefore, timely and effective management of peripheral neuropathy is crucial to reducing the risk of such sequelae.

Clinicopathologic studies have demonstrated that two distinct mechanisms contribute to the development of peripheral neuropathy in EGPA (Nishi et al., 2020). In ANCA-positive patients, peripheral neuropathy results from tissue ischemia and injury caused by vasculitis. Conversely, in ANCA-negative patients, peripheral neuropathy arises from vascular occlusion due to eosinophilia, which leads to neural tissue ischemia and injury. Consequently, in ANCA-negative patients, the rapid elimination of eosinophilia is crucial for the effective treatment of neuropathy. In the case presented, the patient’s persistent peripheral neuropathy, being ANCA-negative, was closely associated with ineffective eosinophil control, and significant symptom relief was achieved following the reduction of eosinophil levels.

Standard treatment for EGPA typically involves the use of corticosteroids and immunosuppressants, which effectively alleviate symptoms in the majority of patients. Corticosteroids offer the significant advantage of rapidly mitigating vasculitis, often within several days. However, prolonged corticosteroid use is associated with considerable adverse events, particularly when dose tapering is not feasible due to uncontrolled or persistent extrapulmonary manifestations. Cyclophosphamide remains the most commonly prescribed immunosuppressant for the management of severe vasculitis as induction therapy. To date, no other medication has demonstrated superior efficacy to cyclophosphamide in this context (Stone et al., 2010; Wechsler et al., 2017).

For patients with refractory neuralgia, rituximab and mepolizumab have demonstrated some efficacy (Thiel et al., 2017; Kai et al., 2022). Rituximab induces B-cell apoptosis, thereby suppressing the production of autoreactive antibodies, and has shown favorable outcomes across various autoimmune disorders (Faurschou and Jayne, 2014). In ANCA-associated vasculitis (AAV), rituximab has been established as non-inferior to cyclophosphamide as an induction agent (Stone et al., 2010). It has since become the standard of care for AAV and is now recommended as a first-line therapy for the management of severe granulomatous polyangiitis (GPA) and microscopic polyangiitis (MPA) (Specks et al., 2013). However, rituximab appears to be more effective in ANCA-positive patients, although ANCA negativity does not preclude its potential benefits (Novikov et al., 2016). Mepolizumab inhibits IL-5 signaling in eosinophils, thereby preventing their activation, recruitment, and accumulation in tissues (Leckie et al., 2000). It has been evaluated in various eosinophilic disorders, including asthma, rhinosinusitis, hypereosinophilic syndrome, and atopic dermatitis (Keating, 2015). Given the proposed central role of eosinophils and elevated IL-5 levels in EGPA, mepolizumab emerges as a logical therapeutic candidate. Evidence of its potential benefit in EGPA has been derived from small open-label pilot studies and case reports. However, prospective studies have not demonstrated its efficacy in addressing severe vasculitis as an induction therapy (Wechsler et al., 2017).

The role of TNF-αin EGPA remains a subject of debate. Blocking TNF-αdemonstrated no effectiveness in the treatment of ANCA-associated GPA (3). However, there is a notable absence of prospective studies evaluating the efficacy of TNF-α blockade in the treatment of ANCA-negative EGPA which is closely linked to asthma, with the majority of EGPA patients having a history of this condition. The asthma population is categorized into “Type-2-high” and “Type-2-low” asthma. About 5%–10% of asthma patients, primarily those with “Type-2-low” asthma, experience severe disease that is refractory or poorly responsive to inhaled corticosteroid therapy. “Type-2-low” asthma is characterized by the absence of Th2 biomarkers and the presence of Th1 biomarkers, such as TNF-α, a proinflammatory cytokine involved in various aspects of airway pathology in asthma (Brightling et al., 2008). Anti-TNF-α therapy has shown improvement in severe refractory cases (Morjaria et al., 2008). In this case, the patient was ANCA-negative, his normal IL-4 levels and elevated IL-6 levels suggested classification into the “Type-2-low” population, explaining their lack of response to inhaled corticosteroids. The rapid and effective response to TNF-α blockade therapy further supported the “Type-2-low” classification, indicating that TNF-α inhibition could be a viable therapeutic option for refractory EGPA in patients with a history of “Type-2-low” asthma. Furthermore, several case reports have highlighted the efficacy of coadministration of TNF-α blockers in the treatment of refractory ANCA-negative EGPA patients (Arbach et al., 2002; Tiliakos et al., 2004).

The limitation of this case is that the patient was not treated with rituximab or mepolizumab. This was due to its relatively high cost and unavailability at our hospital. Another limitation is that we did not adjust the treatment regimen promptly, leading to suboptimal residual limb mobility in the patient. This outcome might have been prevented with more timely evaluations and the use of effective therapeutic agents.

In conclusion, for patients with refractory EGPA with a history of “Type-2-low” asthma, particularly those who are ANCA-negative, TNF-α blockade can serve as an alternative treatment modality. Its rapid onset of action permits a swift evaluation of therapeutic efficacy, thereby minimizing the duration of uncontrolled disease and reducing the risk of irreversible limb dysfunction.

## Data Availability

The raw data supporting the conclusions of this article will be made available by the authors, without undue reservation.

## References

[B1] Arbach O. Gross W. L. Gause A. (2002). Treatment of refractory churg-strauss-syndrome (css) by tnf-alpha blockade. Immunobiology 206 (5), 496–501. 10.1078/0171-2985-00197 12607724

[B2] Brightling C. Berry M. Amrani Y. (2008). Targeting tnf-alpha: a novel therapeutic approach for asthma. J. Allergy Clin. Immunol. 121 (1), 5–10. 10.1016/j.jaci.2007.10.028 18036647 PMC3992375

[B3] Emmi G. Bettiol A. Gelain E. Bajema I. M. Berti A. Burns S. (2023). Evidence-based guideline for the diagnosis and management of eosinophilic granulomatosis with polyangiitis. Nat. Rev. Rheumatol. 19 (6), 378–393. 10.1038/s41584-023-00958-w 37161084

[B4] Faurschou M. Jayne D. R. (2014). Anti-B cell antibody therapies for inflammatory rheumatic diseases. Annu. Rev. Med. 65, 263–278. 10.1146/annurev-med-070912-133235 24160940

[B5] Fei M. Bhatia S. Oriss T. B. Yarlagadda M. Khare A. Akira S. (2011). Tnf-alpha from inflammatory dendritic cells (dcs) regulates lung Il-17a/Il-5 levels and neutrophilia Versus eosinophilia during persistent fungal infection. Proc. Natl. Acad. Sci. U. S. A. 108 (13), 5360–5365. 10.1073/pnas.1015476108 21402950 PMC3069210

[B6] Group. WsGETWR (2005). Etanercept plus standard therapy for wegener's granulomatosis. N. Engl. J. Med. 352 (4), 351–361. 10.1056/NEJMoa041884 15673801

[B7] Kai Y. Yoshikawa M. Matsuda M. Suzuki K. Ohara H. Iguchi N. (2022). Improvement of peripheral neuropathy in a patient with antineutrophil cytoplasmic antibody-negative eosinophilic granulomatosis with polyangiitis by additional mepolizumab. Allergy Asthma Clin. Immunol. 18 (1), 14. 10.1186/s13223-022-00653-7 35183225 PMC8858463

[B8] Kankaanranta H. Ilmarinen P. Zhang X. Adcock I. M. Lahti A. Barnes P. J. (2014). Tumour necrosis factor-α regulates human eosinophil apoptosis via ligation of TNF-receptor 1 and balance between NF-κB and AP-1. PLoS One 9 (2), e90298. 10.1371/journal.pone.0090298 24587316 PMC3938678

[B9] Keating G. M. (2015). Mepolizumab: first global approval. Drugs 75 (18), 2163–2169. 10.1007/s40265-015-0513-8 26603873

[B10] Leckie M. J. ten Brinke A. Khan J. Diamant Z. O'Connor B. J. Walls C. M. (2000). Effects of an Interleukin-5 blocking monoclonal antibody on eosinophils, airway hyper-responsiveness, and the late asthmatic response. Lancet 356 (9248), 2144–2148. 10.1016/s0140-6736(00)03496-6 11191542

[B11] Morjaria J. B. Chauhan A. J. Babu K. S. Polosa R. Davies D. E. Holgate S. T. (2008). The role of a soluble tnfalpha receptor fusion protein (etanercept) in corticosteroid refractory asthma: a double blind, randomised, placebo controlled trial. Thorax 63 (7), 584–591. 10.1136/thx.2007.086314 18245148

[B12] Nishi R. Koike H. Ohyama K. Fukami Y. Ikeda S. Kawagashira Y. (2020). Differential clinicopathologic features of egpa-associated neuropathy with and without anca. Neurology 94 (16), e1726–e1737. 10.1212/WNL.0000000000009309 32217776

[B13] Novikov P. Moiseev S. Smitienko I. Zagvozdkina E. (2016). Rituximab as induction therapy in relapsing eosinophilic granulomatosis with polyangiitis: a report of 6 cases. Jt. Bone Spine 83 (1), 81–84. 10.1016/j.jbspin.2015.04.016 26494587

[B14] Specks U. Merkel P. A. Seo P. Spiera R. Langford C. A. Hoffman G. S. (2013). Efficacy of remission-induction regimens for anca-associated vasculitis. N. Engl. J. Med. 369 (5), 417–427. 10.1056/NEJMoa1213277 23902481 PMC5953195

[B15] Stone J. H. Merkel P. A. Spiera R. Seo P. Langford C. A. Hoffman G. S. (2010). Rituximab Versus cyclophosphamide for anca-associated vasculitis. N. Engl. J. Med. 363 (3), 221–232. 10.1056/NEJMoa0909905 20647199 PMC3137658

[B16] Thiel J. Troilo A. Salzer U. Schleyer T. Halmschlag K. Rizzi M. (2017). Rituximab as induction therapy in eosinophilic granulomatosis with polyangiitis refractory to conventional immunosuppressive treatment: a 36-Month Follow-up analysis. J. Allergy Clin. Immunol. Pract. 5 (6), 1556–1563. 10.1016/j.jaip.2017.07.027 28916432

[B17] Tiliakos A. Shaia S. Hostoffer R. Kent L. (2004). The use of infliximab in a patient with steroid-dependent Churg-Strauss syndrome. J. Clin. Rheumatol. 10 (2), 96–97. 10.1097/01.rhu.0000120897.08819.ac 17043477

[B18] Wechsler M. E. Akuthota P. Jayne D. Khoury P. Klion A. Langford C. A. (2017). Mepolizumab or placebo for eosinophilic granulomatosis with polyangiitis. N. Engl. J. Med. 376 (20), 1921–1932. 10.1056/NEJMoa1702079 28514601 PMC5548295

[B19] Wolf J. Bergner R. Mutallib S. Buggle F. Grau A. J. (2010). Neurologic complications of churg-strauss Syndrome--a prospective monocentric study. Eur. J. Neurol. 17 (4), 582–588. 10.1111/j.1468-1331.2009.02902.x 20050889

